# Facilitators and barriers to effective supervision of maternal and newborn care: a qualitative study from Shinyanga region, Tanzania

**DOI:** 10.1080/16549716.2021.1927330

**Published:** 2021-06-20

**Authors:** Tumaini Mwita Nyamhanga, Gasto Frumence, Anna-Karin Hurtig

**Affiliations:** a Department of Development Studies, Muhimbili University of Health and Allied Sciences, Dar Es Salaam, Tanzania; b Department of Epidemiology and Global Health, Umeå University, Umeå, Sweden

**Keywords:** Facilitators and barriers, supportive supervision, integrated supportive supervision, maternal and newborn care, quality

## Abstract

**Background**: Despite routine supportive supervision of health service delivery, maternal and newborn outcomes have remained poor in sub-Saharan Africa in general and in Tanzania in particular. There is limited research evidence on factors limiting the effectiveness of supportive supervision in improving the quality of maternal and newborn care.

**Objective**: This study explored enablers of and barriers to supportive supervision in maternal and newborn care at the district and hospital levels in Shinyanga region in Tanzania.

**Methods**: This study employed a qualitative case study design. A purposeful sampling approach was employed to recruit a stratified sample of health system actors: members of the council health management team (CHMT), members of health facility management teams (HMTs), heads of units in the maternity department and health workers.

**Results**: This study identified several barriers to the effectiveness of supportive supervision. First, the lack of a clear policy on supportive supervision. Despite the general acknowledgement of supportive supervision as a managerial mechanism for quality improvement at the district and lower-level health facilities, there is no clear policy guiding it. Second, limitations in measurement of progress in quality improvement; although supportive supervision is routinely conducted to improve maternal and newborn outcomes, efforts to measure progress are limited due to shortfalls in the setting of goals and targets, as well as gaps in M&E. Third, resource constraints and low motivation; that is, the shortage of resources – CHMT supervisors, health staff and funds – results in irregular supervision and low motivation.

**Conclusion**: Besides resource constraints, lack of clear policies and limitations related to progress measurement impair the effectiveness of supportive supervision in improving maternal and newborn outcomes. There is a need to reform supportive supervision so that it aids and measures progress not only at the district but also at the health facility level.

## Background

### A quest for quality maternal and newborn care

Preventable maternal and neonatal mortality is not simply an issue of access to health services during pregnancy and childbirth, but also, importantly, access to services of acceptable quality [1,2]. Concerns about the poor quality of maternal and new-born care have been reported in low and lower-middle-income countries [3,4]. It is argued that whereas there has been improvement in access and utilization of maternal and neonatal health care in those countries, mortality rates remain high [3]. Consequently, huge gaps exist between low and high-income countries with respect to maternal and neonatal deaths. For instance, low-income countries have an average lifetime maternal mortality of 1 in 38 compared to 1 in 3700 for high-income countries [5]. Moreover, 99% of 2 million annual neonatal deaths occur in low and middle-income countries (LMICs) [6]. The high levels of mortality are attributed to poor quality of care in the health facilities [3], characterized by overcrowding and understaffing, with inappropriate management of complicated deliveries and untimely response to emergencies [7]. It is therefore evident that poor quality of care in the LMICs is a hindrance to achieving Sustainable Development Goal 3: ‘ensure healthy lives and promote well-being for all at all ages’ [8]; mere access to health facilities is necessary but not sufficient [9].

Likewise, country-specific studies have reported poor quality of obstetric care in Tanzania. For instance, Mselle et al [10] revealed that women who had obstetric fistula experienced a lack of support, neglect, as well as physical and verbal abuse by the service providers [6]. Another study conducted in Tanzania at Muhimbili National Hospital by Kidanto et al. [11] also identified suboptimal care in about 80% of audited cases, out of which about 50% were found to be the likely cause of an adverse perinatal outcome. Inadequate maternal and foetal monitoring during labour were the main suboptimal factors.

Interventions to address the poor quality of maternal and newborn care in Tanzania have involved advocacy for skilled birth attendance, availability of skilled personnel, medical supplies, and infrastructure improvements [12,13]. Relatively little attention has been paid to how governance and management interventions can improve the quality of maternal and newborn care; specifically, how supportive supervision (SS) can contribute to improving the quality of maternal and newborn services has not received sufficient attention [14–18]. These studies suggest that although SS is implemented routinely in Tanzania, limited attention has been put into enhancing the effectiveness of its implementation.

### The role of supportive supervision in the quality of maternal and newborn services

Supervision of healthcare services has evolved from a traditional approach – which focuses on inspecting facilities and ‘controlling’ individual performance – to SS which seeks to guide, help, train and encourage staff to improve their performance to provide high-quality healthcare services [6,7,19]. Regular SS can thus be the catalyst for improvement in both performance and service quality at various levels throughout the health system [19]. Such improvement through SS is carried out by defining desired performance, setting standards, assessing performance, finding causes for any performance gaps, selecting and implementing steps to fill those gaps, and monitoring and evaluating progress [20]. Two approaches are variably practiced across the world: integrated supportive supervision (ISS) and technical supportive supervision (TSS) [21]. ISS is concerned with the supervision of systems and services, and the focus is on managers at different levels. TSS is more concerned with the supervision of care quality and the focus is on practitioners. TSS is hereby defined as ‘the formal provision, by approved supervisors, of relationship-based education and training that is work-focused and which manages, supports, develops, and evaluates the work of colleagues [22].

### Supportive supervision in Tanzania

Tanzania predominantly practices ISS, which is carried out at various levels, as depicted in the National guideline for Internal Supportive Supervision and External Hospital Performance Assessment (EHPA) for Regional Referral Hospitals [23]. The supervisory roles at these levels are described as follows: at the national level the Ministry of Health, Community Development, Gender, Elderly and Children (MoHCDGEC) conducts managerial SS for national, zonal referral, specialized hospitals, and regional health management teams (RHMTs). At the regional and council levels, managerial SS is the responsibility of the RHMTand is conducted quarterly. That is, the RHMT oversees the deployment and implementation of health policies and guidelines by the regional referral hospital management team (RRHMT) and council health management team (CHMT). At the council level, the council is the focal point for the promotion of health policy and health interventions, and the CHMTs supervise systems and services at district hospitals, health centres and dispensaries on a quarterly basis [23].

Effective SS requires a functioning system for monitoring and evaluation (M&E). The M&E in Tanzania’s health system is characterized by the implementation of a Health Management Information System (HMIS) involving use of registers to document information about patient visits, diagnoses, treatment, and outcomes, among others, at the health facilities [24]. The monthly aggregate summaries are then recorded on summary forms and submitted to the districts which eventually enter the same into the MoHCDGEC electronic system known as District Health Information System 2 (DHIS2) [25]. The DHIS2 software is used to manage and analyze aggregate data reported by health facilities, thereby contributing to improving service delivery [25].

Improvement of maternal and newborn services is one of the top priority areas targeted by SS [15,23]. SS is a mechanism for monitoring and improving the quality of maternal and newborn healthcare delivered by health workers, but supervisory practices in Tanzania have had a limited impact in this regard [26–33], as evidenced by poor maternal and newborn outcomes attributable to weaknesses in service provision during pregnancy and childbirth [31–33].It is not clear why there are weaknesses in service provision during pregnancy and childbirth despite the existence of routine SS mechanism. There is limited research evidence on systemic enablers of and barriers to effective SS for maternal and newborn care in Tanzania’s district health system. Therefore, the aim of this study was to explore enablers of and barriers to supportive supervision in maternal and newborn care at the district and hospital levels.

### Conceptual framework

The conceptualization of this study was informed by two theories: Lo Locke and Latham’s Goal-setting Theory and the Theory of Constraints (ToC) [34–36]. The two theories informed identification of the research gap, the setting of research questions and objectives, and the development of an interview guide for data collection. Locke and Latham’s Goal-setting Theory states that setting a goal is key to quality improvement. In the context of this study, it is argued that when institutional and staff goals are set, the supervisor and supervisees work harder to achieve them. Other facilitative factors include but are not limited to: a supportive relationship between supervisor and supervisee; feedback on progress; and supportive contextual factors such as resources, training, and favourable policies. The ToC states that every system has at least one constraint, which is anything that limits the system from achieving higher performance in terms of its goal. The constraints to SS may include a heavy workload, limited training for both healthcare workers and members of the supervision teams, unfavourable policies, and a less supportive regulatory environment. The existence of constraints or barriers represents an opportunity for improvement. In the light of this conceptual framework, the study set out to answer the following question: ‘What are the facilitators and barriers to effective supportive supervision of maternal and newborn care in Shinyanga Region, Tanzania?’

## Methods

### Design

This study employed a qualitative case study design. This design enabled the researchers to explore contextual barriers to supportive supervision using interviews and document reviews. Because of its flexibility and comprehensiveness, the case study design enabled deep exploration of broad facilitators and barriers to supportive supervision – as depicted in the conceptual framework [37].

### Study site and justification

The target area, Shinyanga, is one of the regions which had higher maternal mortality than the national average (432/100,000 live births) in 2015 [38]. Shinyanga is one of eight regions – alongside Kagera, Geita, Simiyu, Mwanza, Mara, Tabora, and Kigoma – with the poorest reported maternal newborn and child health (MNCH) indicators in Tanzania [39]. Shinyanga is located in the northwestern part of Tanzania and consists of five Councils: three district councils (DC; Shinyanga, Kahama, and Kishapu); one municipal council (MC; Shinyanga), and one town council (TC; Kahama) [40].

### Data collection

Two districts were involved in this study, from October – December 2019. In each selected district, the district hospital, two health centres and two dispensaries were selected on the basis of poor and better maternal and newborn health indicators – particularly on the status of maternal and neonatal mortality. As indicated in Table 1, in each district, six key informants were purposefully recruited. These included officers dealing with maternal and newborn care from members of the CHMTs and HMTs and heads of units in the maternity department and those in charge of health centres and dispensaries. In addition, in each district members of the CHMT and HMT were invited to take part in focus group discussions (FGDs). There were two FGD sessions in each of the two districts – one for CHMT members and another for HMT members. Each FGD consisted of 6 members, making a total of 24 FGD participants.

**Table 1.** Purposefully sampled participants from the two districts

A	**Key Informants**	**Number**
	District Medical Officer	2
	District Reproductive and Child Health Coordinator	2
	Medical Officer In-Charge of District Hospital	2
	Head of Reproductive and Child Health services of the District Hospital	2
	In-charge of Health Centre	2
	In-charge of Dispensary	2
**B**	**Focus Group Discussion Participants**	
	Members of Council Health Management Team (CHMT) – 6 members per district (x 2)	12
	Members of Hospital Management Team (HMT) – 6 members per district (x 2)	12

Data were collected through key informant interviews, FGDs, and document reviews. The interviews were deemed appropriate as they involved one-on-one interaction with a wide range of officers who are informed about supportive supervision. Moreover, the interviews were complemented with FGDs which helped to uncover issues that initially may not have been considered by individual interviewees. Interview and FGD guides were developed and used to conduct one-to-one interviews and group discussions, respectively. The guides consisted of broad questions on facilitators and barriers to SS, reflecting the constructs in the conceptual framework. Whereas the interviews took place in the office of the respective key informant, focus group discussions were conducted in the seminar/conference room commonly used for meetings at the district headquarters or at the health facility. Both the interviews and FGDs were audio-recorded and privacy was ensured during the process. Document reviews involved reading the national supportive supervision guideline and quarterly and annual supervision reports – provided by the office of the District Medical Officer – with a view towards identifying success stories and challenges encountered.

### Data analysis

Thematic analysis, following both deductive and inductive approaches, was used to analyze the collected focus group and interview data. The deductive approach was applied from the data collection stage, during which FGD and interview guides were informed by the conceptual framework that was developed from the select theories (moving from theory to data), and subsequent inductive generation of themes was done in the context of the predetermined constructs of the framework. The use of an inductive approach sought to ensure that the emerging themes were strongly linked to the data, rather than being imposed by the researcher [41,42]. The audio-recorded FGDs and interviews were transcribed verbatim and translated from Kiswahili to English. The analysis was done manually in three stages: line-by-line coding of field notes and transcripts (i.e. development of concepts; data were broken into discrete parts to expose the thoughts and meanings they contained); examination and interpretation of codes into descriptive themes; and condensation of descriptive themes into more abstract analytical themes.

Likewise, document review involved two main steps. Firstly, a scheme for organizing the content was developed. It reflected the constructs in the conceptual framework. Secondly, reports were read and information therein related to the constructs was extracted in summary and categorized accordingly.

## Results

The thematic analysis generated five main themes describing enablers to and barriers to effective supervision of maternal and newborn care in the selected health facilities, which are presented in greater detail below.

### Enablers of supportive supervision by the CHMTs

The enablers include CHMTs’ commitment to improving maternal and newborn outcomes and budgeting for supervision.

### High level of commitment to improving maternal and newborn outcomes

It was reported that a high level of commitment of the CHMT to reducing incidents of maternal and neonatal deaths in the district serves as a driving force for the adoption of supervision of service delivery systems as a facilitative approach. One of the key informants thus said:

As managers of health care in the district, the reduction of maternal and neonatal deaths is our topmost priority. It is at the center of our responsibilities. Thus we make sure that services are running. We conduct supervision to identify challenges and address them accordingly. (KI#9, CHMT member)

Further that the implementation of supervision of service delivery systems is made possible through teamwork arrangement. That is, CHMT members move as a team when paying supervisory visits and each member inquires about the status of maternal and newborn care in the context of her/his respective professional domain; followed by coordinated in-depth problem analysis and finding of solution(s). One of the CHMT members fittingly said:

When it comes to field visit for supervision everybody joins the team and leave whatever pending activities – so that all dimensions of maternal and newborn care are addressed on a single visit. (KI#7, CHMT member).

### Budgeting for supervision

It was reported that due to its importance supervision is included in the comprehensive district health plans and budgeted for, making it sustainable. Further that the districts do budget for fuel, vehicle maintenance, and supervision allowance. Moreover, it was pointed out that when supervisors receive supervision allowance they get motivated.

Every year we budget for supervision as it is a major component in the CCHP (Comprehensive Council Health Plan). We budget for fuel, vehicle maintenance, and supervision allowance. (KI#3, CHMT member).

### Barriers to supportive supervision

The identified barriers to supportive supervision are: lack of clear SS policy; limitations in measurement of progress in quality improvement; and resource constraints resulting in irregular supervision and low motivation.

#### Lack of a clear policy on supportive supervision

It was found that despite a general appreciation of the importance of SS for quality improvement at the district and lower-level health facilities, there was no clear policy guiding it. As a result, when responding to what SS entails, it became apparent that CHMT supervisors focused more on assessing facility-level service provision and less on individual staff performance. This is also reflected in the national SS guideline, the content of which focuses more on administrative and logistical aspects and significantly less on clinical competence. That is, the areas of focus of the national SS guideline include: leadership and governance; annual planned activities; financial status; human resource for health; health commodities and medical supplies; physical assets, hospital environment; and services provision and quality. The latter [services provision and quality] is not directly about improving clinical competence of the practitioners, it is on the health facility’s readiness of service provision, availability of standard operating procedures, client satisfaction, safety issues, quality improvement (QI) implementation structures, and their functionality. It is therefore clear that the national SS guideline which guides Regional and District level supervisory authorities has a predominant focus on ISS versus TSS. Nevertheless, facility-level SS appears to be more clinical but less formal, as there is no guideline. Consequently, its rigour varies from one facility or unit to another, depending on the capability and eagerness of the manager. This was well expressed by one of the key informants:

At the ward level, supervision depends on the competence and enthusiasm of the person in charge. If she or he is competent enough s/he would be enthusiastic to do regular ward rounds, going bed to bed reviewing care provided to mothers and newborns, and giving constructive comments. Two years ago, we had a person in charge who was not very competent: she rarely conducted ward rounds. On those occasions, she was challenged by the subordinates and did not like it. (KI#4, Midwife).

#### Limitations in measuring quality improvement progress

It was noted that although SS is routinely conducted to improve maternal and newborn outcomes, efforts to measure progress are limited due to shortfalls in setting goals and targets, as well as gaps in monitoring and evaluation (M&E). On limitations in setting goals and targets, it was found out that although the district health department does set goals and targets, the hospitals, health centres, and dispensaries do not set their own. The health facilities within a particular district are informed of the district-level goals and targets and work towards achieving them. One of the hospital managers said:

The goals and targets for addressing challenges in maternal and newborn care are set at the district level by the CHMT. The role of a hospital like ours is to work hard and eventually improve the district situation. (KI#1, Hospital Manager).

In connection with the lack of goals and targets, it was noted that M&E is non-existent at the health-facility level and only partially present at the district level. It was reported that districts use the district health information system (DHIS2) to conduct situational analysis that identifies maternal and newborn problems and then sets goals and targets towards addressing them. DHIS2 summarizes the most important information, including scorecards which provide rate performance indicators by colour (red for poor, yellow for fair and, green for best). Nevertheless, it was admitted there are problems with M&E. Once goals and targets are set, they are not frequently monitored to ensure action is taken or the situation improves. Usually, review is done once a year at the district level. Consequently, data generated from the facilities are not optimally used to improve the quality of maternal and newborn care in the short term. One of the district medical officers acknowledged:

The only challenge we have is M&E. We do not have experts in this area so, more investment is required. (KI#9, DMO).

Another respondent added that because of limited monitoring, it is difficult to measure the achievement of SS in improving the quality of maternal and newborn care in the health facilities:

We have data in the DHIS2 which we use when writing district health plans, and supervisors from the district follow up to see that data is correctly collected and entered in the system; but they rarely use the data to evaluate progress made by the reporting health facilities in improving maternal and newborn outcomes between supervisory visits. (KI#11, CHMT member).

#### Resource constraints resulting in irregular supervision and low motivation

A review of annual and/or quarterly reports showed that SS is constrained by a shortage of resources, including CHMT supervisors, health staff, and funds. Key informants further elaborated that there are few supervisors (CHMT members) relative to the number of health facilities, which delays the exercise of SS. Consequently, to increase coverage, maternal and newborn care supervisors often pay more attention to facility-level service provision and ask questions on the availability of supplies, number of deliveries, and maternal mortality figures, as well as proper feeding of data into the DHIS2. Coaching and mentorship towards improving individual staff performance were reported to be rare.

The limited number of supervisors and the transport problem in a rural district like this compel us to make sure that we cover at least two facilities per supervisory visit. It is like a rush. As such, clinical supervision is either not done or done insufficiently due to time constraints. (FGD 3, CHMT member #16).

Less attention to coaching and mentorship was also attributed to limited technical capacity. It was argued that, although CHMT supervisors are basically health professionals, some of them have not been practicing direct patient care for quite sometime and have literally lost clinical skills. One of the CHMT members was honest enough to admit this limitation:

It is true that the CHMT comprises people trained as doctors, nurses, pharmacists [and] laboratory scientists but honestly, they have lost touch with clinical work because of managerial duties here at the district. That is why when they go to supervise, say, a hospital, they avoid engaging in clinical aspects. I think there is a need for a system whereby supervisors will continuously update their clinical skills. (FGD 4, CHMT member #22).

Similarly, it was reported that the shortage of healthcare staff at the health-facility level constrains SS as it renders individualized coaching and mentorship of doctors and midwives by CHMT supervisors impractical:

Maternity is the busiest and most sensitive department. For instance, with the serious staff shortage we have here, the labour ward is often staffed with 2–3 midwives per shift. The heavy workload makes coaching and mentorship by CHMT supervisors not feasible. (KI#10, Labour ward person in charge).

On the inadequacy of financial resources, it was reported in both studied district councils that budgetary constraints affected the coverage and effectiveness of SS. Although both councils reported that they set aside funds for SS, the CHMTs experience budgetary constraints in two ways: the approved amount falls short of the requested amount by far, and the approved funds are disbursed late – for instance, funds for the first quarter may be received in the second or third quarter and by the end of the financial year not all approved funds are actually received by the respective CHMT. This bottleneck affects the timely availability of funds for vehicle maintenance, the fuelling of the vehicles, and staff allowances. As a consequence, some of the scheduled supervisory visits are not carried out or are delayed. That lack of money particularly affected remote rural health centres and dispensaries, which were visited once a year instead of quarterly – thereby fragmenting supervision. The effect of financial constraints was succinctly put by one of the respondents:

We are limited by the budget. We get just a fraction of our actual needs for supportive supervision in this district, often late. There are some distant dispensaries that we visit once a year instead of quarterly visits because of financial limitations. (FGD 1, CHMT member #2).

Regarding low motivation, it was reported that most members of CHMT, like other health staff, feel demotivated in the course of carrying out supervisory functions. The respondents attributed their demotivation to low salaries and limited incentives relative to their demanding managerial duties. This observation was succinctly put by one of the CHMT members:

Most CHMT members – particularly those of us who are in the rural districts – do not work to their maximum capacity. We have low salaries, and yet we are expected to serve some of the very remote dispensaries. There is a high level of work dissatisfaction here. (FGD 3, CHMT member #17).

## Discussion

This study explored the enablers of and barriers to effective supervision of maternal and newborn care in Shinyanga region in Tanzania. From the study findings, it appears the enablers include: high level of CHMTs’ commitment to improving maternal and newborn outcomes and budgeting for supervision. On the other hand, barriers that affect the effectiveness of SS, include: lack of a clear SS policy; limitations in measurement of progress in quality improvement; and resource constraints resulting in irregular supervision and low motivation. The implication of these barriers is that Tanzania’s health system has had a predominant focus on ISS at the expense of TSS. Optimum contribution of supportive supervision to quality improvement requires implementation of both ISS which involves supervision of administrative and logistical systems and TSS which is concerned with supporting practitioners to improve their performance. Implementing ISS despite the identified barriers is a reflection that it is a basic mode of supervision requiring relatively less organizational and resource capacity, compared to TSS. Discussion of the specific findings follows.

### Regarding enablers

The study found out that a high level of commitment of the CHMT to reducing incidents of maternal and neonatal deaths in the district serves as a driving force for supportive supervision. This finding suggests that improving maternal and newborn health is a top national agenda that district-level health managers are tasked to implement to justify their existence. The central government through the two Ministries, namely: the President’s Office – Regional Administration and Local Government (PO-RALG) and the MoHCDGEC expect the CHMT to play a key role in the implementation of the health policy that prioritizes maternal and child health. The role of the high level of commitment of the district health management has been similarly reported in Ethiopia [43].

Furthermore, the study revealed that due to its importance supervision is included in the comprehensive district health plans and budgeted for, making it sustainable. This finding suggests that supportive supervision is highly regarded as a key managerial mechanism for improving maternal and newborn outcomes. Inclusion in the district health plans and budgets has sustained it over the years despite some barriers as found out in this study. This finding is corroborated by that reported in the Gambia by Conn et al [44,45,46,47] who found out that allocation of the budget was vital for successful and sustainable supportive supervision.

On the barriers, the study revealed that there is no clear policy guiding SS in the country. Whereas SS seeks to improve the quality of maternal and newborn care, the focus of the intervention seems to be less comprehensive in scope and thus compromises its effectiveness – that is, the focus of SS is more external rather than internal. This means that the hospitals and lower-level health facilities receive quarterly supervision from teams from the district headquarters (CHMT teams), per the national supervision guideline, which also stipulates areas of concentration: leadership and governance, annual planned activities, financial status, human resources for health, health commodities and medical supplies, services provision and quality, physical assets and hospital environment [25]. A critical review of the national supervision guideline and analysis of the interviews suggests that the supervision of hospitals by CHMTs concentrates more on assessing systems by reviewing records and has an insufficient focus on direct quality elements such as staff performance. The guideline is virtually silent on what should be happening at the local level involving management structures at the hospital, maternity department and, ward levels on a daily, weekly, or monthly basis.

This study revealed that although the districts set goals and targets for improving maternal and newborn outcomes through the CHMTs, the health facilities do not set goals or targets. This affects the achievement of quality improvement because it becomes difficult to measure the progress being made. Experts in M&E argue that health facilities can only improve what they can measure [48]. When the goals and targets are set at the health-facility level, they serve as a driving force that pushes the supervisor and supervisee(s) to work harder to achieve them [48], as propounded by Locke and Latham’s Goal-setting Theory [34]. Lack of this driving force in the supervision of maternal and newborn care might partly explain the insignificant reduction of maternal mortality in the past decade, despite the existence of routine SS [49]. This is in line with another study, which found empirically that weakness in setting goals and targets was linked with limited improvement in medical management in Ugandan public health facilities [50].

Furthermore, this study revealed that although district-level goals and targets are set, they are not frequently monitored for the purpose of taking action for improvement. This points to the fact that M&E systems in healthcare either are not well developed or are, at best, fragmented. The district targets are set and achievements evaluated annually, but without or with limited monitoring. This suggests that districts do not continuously analyze DHIS2 data about maternal and newborn outcomes over time and use the outputs to determine corrective actions. It is no wonder that the health facilities in the study setting do not set institutional, departmental and ward-level targets, and that the data generated by these health facilities and fed into DHIS2 are not optimally used to improve maternal and newborn outcomes at the local level. These findings are in agreement with those reported by Holvoet and Inberg [51] who assessed health sector M&E systems in Uganda and Rwanda and came to a similar conclusion about their fragmentary nature. Given the high level of national attention put on improving maternal and newborn outcomes [32], the need for well-functioning M&E systems both at the district and health-facility levels cannot be overemphasized. Therefore, the current study enriches Locke and Latham’s Goal-setting Theory by adding that the setting of goals and targets should happen at both managerial and service delivery levels, underscoring the need for local ownership of the quality improvement process. That is, practically, setting goals and targets at both the district and facility levels will serve two purposes. One, it will stimulate local use of data for local improvement of quality of care. Two, it will facilitate measurement of progress made by health facilities in course of the supervisory visits conducted by the district health managers.

Besides, this study found that SS is constrained by a shortage of resources – CHMT supervisors, health staff, and funds – as rightly stated in the ToC [36]. Regarding the shortage of supervisors, the findings suggest that CHMTs have multiple managerial responsibilities at the district level, besides conducting supervisory visits. This reduces their attention to the details stipulated in the guideline and might also be affecting the depth of feedback from the supervisors to the supervisees as time for meaningful support becomes limited. A similar study conducted in Ghana by Bornenberger et al [52] reported that a shortage of district health managers led to inefficiency in their duties including supervision. Concerning staff shortages at health facilities, the findings imply that the few available doctors and midwives struggle to cover service provision in the antenatal, delivery and postnatal units. There is very limited or no time for individual coaching and mentorship. These findings are corroborated by those reported in Malawi by Bradley et al [28] who found out that staff were so overburdened that supervisors had to assist supervisee to accomplish routine tasks rather than actually supervising. This study found that budgetary constraints affect the coverage and effectiveness of SS. This finding points to the fact that the insufficiency of and delayed disbursement of funds for supervisory visits makes supervision infrequent and less rigorous, particularly in remote health centres and dispensaries. Moreover, limited funding leads to unreliable means of transport because lack of or delayed funding affects the maintenance and fuelling of vehicles, contributing to irregular supervisory visiting of health facilities. Overall, resource constraints might be compelling districts to conduct supervisory visits without paying attention to the quality of the supervision. Several studies [15,53–55] have similarly reported that a shortage of resources renders SS less effective in sub-Saharan Africa.

In the same context of resource shortages, it was found that supervisors – like other health workers – have low motivation. This finding suggests that the CHMTs conducting supervision of maternal and newborn care are distracted, as they have grievances about low salaries and limited incentives. Low motivation among health managers can have a negative impact on the entire health system as they develop an attitude of indifference to quality improvement. Similar findings have been reported elsewhere in sub-Saharan Africa and other developing regions [52,56–58]. It is important to pay more attention to motivating the managers and staff working in the rural districts, as they have a higher burden for supporting improvements that may reduce maternal mortality. Additionally, motivation is more important in this era of the COVID-19 pandemic when staff are torn between saving mothers’ lives and protecting themselves from acquiring the highly infectious and fatal disease.

### Trustworthiness of the findings

The trustworthiness of the results was secured in several ways, such as pretesting, prolonged engagement, member checks and peer debriefing [59]. Interview guides were pretested to understand how well they elicited responses to the study objectives. The data collection period was planned such that the researchers had time for reflection between field visits and were therefore able to conduct preliminary analyses that guided their subsequent data collection. A member check technique was applied during key informant interviews whereby the interviewer restated or summarized the information from the respondent to ensure that what was heard was in fact correct. Peer debriefing involved a review of the coding process by a colleague to see that final themes were grounded in the data. The paper also benefited from triangulation, as data were synthesized from all categories of in-depth interviews, FGDs and document reviews.

Nevertheless, the study might have suffered social desirability bias in the sense that since respondents were health managers responsible for implementing SS, they might have underreported barriers to effective SS for fear that their own answers may have negative implication on the evaluation of their performance. This effect was mitigated by assuring the respondents of anonymity and confidentiality.

## Conclusions

Despite signals of SS policy intent and the existence of routine supervisory practices, their meaningful contribution to improving the quality of maternal and newborn care is deeply challenged. That is, in spite of some enabling factors, there are substantial systemic barriers – lack of a clear SS policy; limitations in measurement of progress in quality improvement; and resource constraints – that impair the contribution of SS to the improvement of maternal and newborn outcomes. These barriers make it difficult to measure the contribution of SS to quality improvement. Hence, there is a need to reform SS so it engages in measuring progress not only at the district but also at the health facility level. That is, SS should incorporate M&E in its implementation. Moreover, SS in Tanzania should go beyond integrated supportive supervision (ISS), which focuses on healthcare managers, to emphasize technical supportive supervision (TSS), which engages in direct assessment of staff performance.

## Paper context

Supportive supervision (SS) is key to improving quality of health care. Previous studies on barriers to SS have been narrowly focused on resource limitations. Apart from resource constraints, this study has revealed that poorly implemented M&E systems impair the contribution of SS to the improvement of maternal and newborn outcomes. There is need for health facilities to set local goals and targets toward improving maternal and new-born outcomes, which should be monitored quarterly by supervisors.
